# Augmentation in fragility fractures, bone of contention: a systematic review

**DOI:** 10.1186/s12891-022-06022-0

**Published:** 2022-12-02

**Authors:** Eleonora Piccirilli, Ida Cariati, Matteo Primavera, Rebecca Triolo, Elena Gasbarra, Umberto Tarantino

**Affiliations:** 1 Department of Orthopaedics and Traumatology, “Policlinico Tor Vergata” Foundation, Viale Oxford 81, 00133 Rome, Italy; 2 Department of Clinical Sciences and Translational Medicine, “Tor Vergata” University of Rome, Via Montpellier 1, 00133 Rome, Italy

**Keywords:** Osteoporosis, Bone fragility, Augmentation techniques, Elderly, Fragility fractures, Fracture healing, Augmentation strategies

## Abstract

**Background:**

Osteoporosis is a complex multifactorial disease characterized by reduced bone mass and microarchitectural deterioration of bone tissue linked to an increase of fracture risk. Fragility fractures occur in osteoporotic subjects due to low-energy trauma. Osteoporotic patients are a challenge regarding the correct surgical planning, as it can include fixation augmentation techniques to reach a more stable anchorage of the implant, possibly lowering re-intervention rate and in-hospital stay.

**Methods:**

The PubMed database and the Google Scholar search engine were used to identify articles on all augmentation techniques and their association with fragility fractures until January 2022. In total, we selected 40 articles that included studies focusing on humerus, hip, spine, and tibia.

**Results:**

Literature review showed a quantity of materials that can be used for reconstruction of bone defects in fragility fractures in different anatomic locations, with good results over the stability and strength of the implant anchorage, when compared to non-augmented fractures.

**Conclusion:**

Nowadays there are no recommendations and no consensus about the use of augmentation techniques in osteoporotic fractures. Our literature review points at implementing the use of bone augmentation techniques with a specific indication for elderly patients with comminuted fractures and poor bone quality.

## Background

Osteoporosis is a systemic skeletal disease characterized by a decreased bone density and a deterioration in bone quality (microarchitectural changes), leading to compromised bone strength and an enhanced risk of fractures [1]. The prevalence of osteoporosis increases with age and is more common among women than men [2]. The most recent data estimate that one in three women over the age of 50 and one in five men over the age of 65 will suffer a bone fragility fracture, confirming it as one of the major problems facing health systems worldwide [3]. Osteoporotic fractures occur when a mechanical stress applied to the bone exceeds its strength. The most frequent fracture sites are the proximal femur, the vertebrae, the proximal humerus, and the distal radius [4]. According to the World Health Organization (WHO), fragility fractures result from low-energy trauma due to mechanical forces equivalent to a fall from a standing height or less, which would not ordinarily cause a fracture. It is now believed that skeletal fragility requires both decreased bone density and poor bone quality, defined as alterations in bone architecture, bone geometry, and the material properties of the microstructural constituents as well as the presence of microdamage [5–7].

According to numerous evidences, the pathogenesis of osteoporosis is complex and probably affects bone strength depending on multiple interactions between local and systemic regulators of bone cell function, such as osteoblasts, osteoclasts and osteocytes [8, 9] and on the reduction in cross-linking between collagen fibers, the decrease in structural horizontal trabeculae and the thinning of vertical trabeculae [10–12]. In this complex fragility framework, it is well known that fragility fractures after a surgical treatment could lead to a terrible complication such as implant failure: for proximal femoral fracture the implant failure rate is estimated up to 6% [13, 14]. For this reason, elderly osteoporotic patients who generally present with poor bone quality, comminuted and unstable fracture patterns are at increased risk of early mechanical failure and therefore show an indication for augmentation [15]. Fixation augmentation techniques are defined as any surgical procedure that increases implant stability. They include a variety of biological and orthobiological materials, such as polymethylmethacrylate (PMMA), bone grafts, calcium phosphate ceramics including blocks, cements and coatings, and modified implants [16]. Thermal damage and cement leakage are the two most common complications [17, 18]. According to various studies, augmentation techniques are safe; however, possible outcomes include stroke, heart attack, embolism or infection are all possible outcomes [19, 20]. When using cement, fragility fractures have a lower complication rate than total joint replacement, as the cement is injected at a lower pressure [20]. Furthermore, among the potential benefits of augmentation, lower re-intervention rates and a reduction in total hospital stay have been observed [21].

To date, there is no global consensus on the possible indications for using augmentation techniques in fractured patients. Therefore, in this systematic review we would like to investigate whether there is unambiguous evidence about the use of augmentation techniques in different sites of fragility fractures occurring in the elderly population affected by osteoporosis.

## Methods

### Source of studies and search strategy

**Fig. 1:**
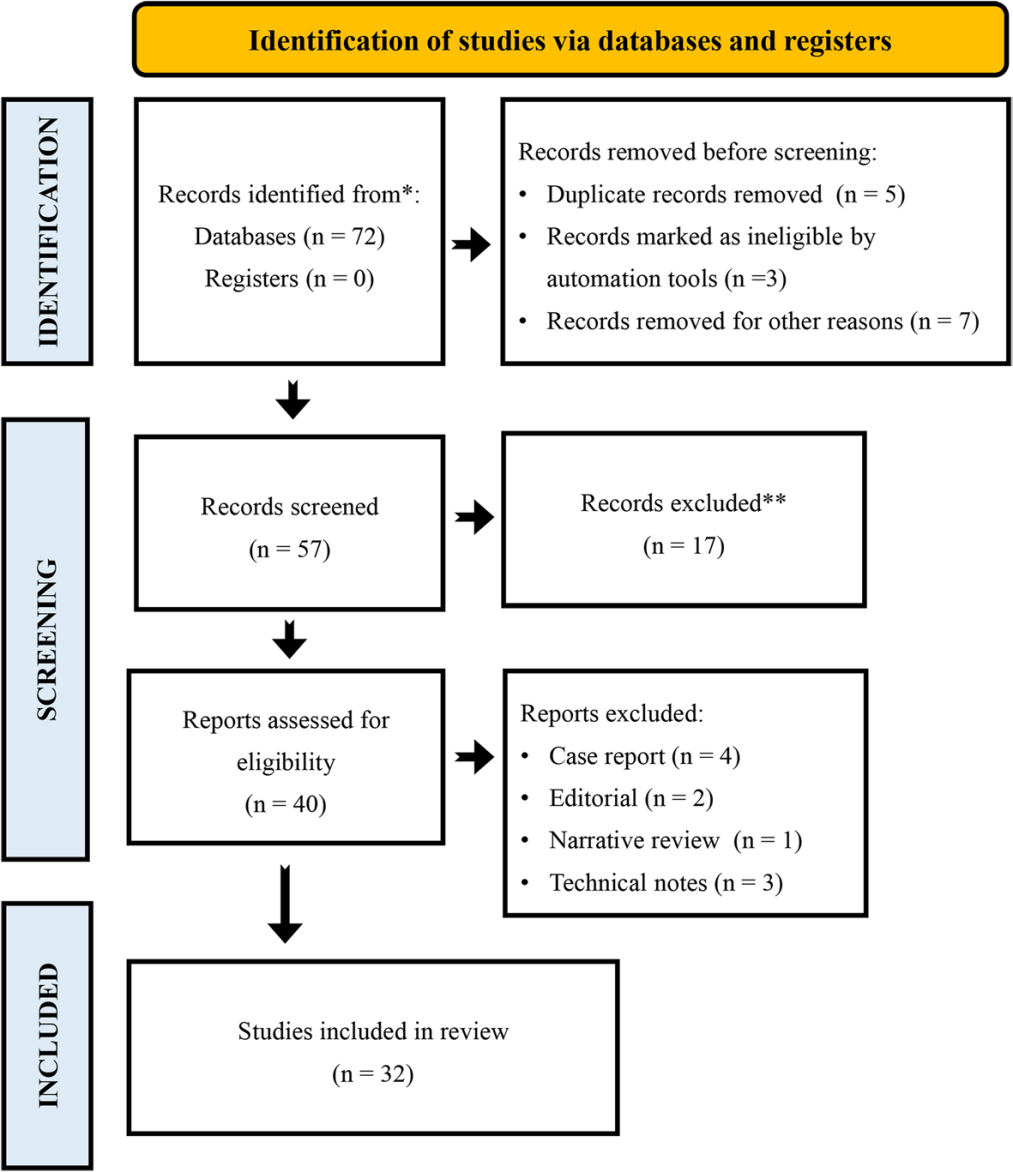
PRISMA 2020 flow diagram of the literature search and selection process of the included studies

A systematic review was conducted following the recommendations of the Preferred Reporting Items for Systematic Reviews and Meta-Analyses (PRISMA) (Fig. 1). We performed a search in the last twenty years up to January 2022 on the PubMed and Google Scholar electronic databases of English-only papers associating augmentation techniques and fragility fractures in osteoporotic patients. The search strategy covered all the augmentation techniques, excluding pharmacological augmentation concepts, and their association with fragility fractures. In the search strategy, we used various combinations of the following key terms: “augmentation techniques”, “fragility fractures”, “humerus”, “spine”, “pelvic ring”, “distal tibia”, “ankle”. Case reports, editorials, technical notes, and narrative review articles were excluded.

### Study selection and eligibility criteria

The search and evaluation of the articles was carried out independently by two orthopedics, while a researcher experienced in systematic reviews resolved any doubts. As previously described, each reviewer read the abstracts of all articles, selecting the relevant ones according to specific inclusion and exclusion criteria and then comparing the results with the other reviewer [22]. After two weeks, the same studies were read again to establish the researchers’ agreement on the selection. No disagreement was observed among the researchers.

### Data collection

One reviewer extracted the data from the full-text articles to Excel spreadsheet structured tables to analyze the study in a descriptive fashion. The second researcher independently double checked the extraction of primary data from all the articles. Doubts and inconsistencies were followed and solved by discussion. The following information was extracted from articles: type of fracture, type of augmentation technique, population, methods, and results. We selected a total of 32 articles including studies focusing on fragility fractures at different anatomical sites: humerus (4 studies), hip (13 studies), tibia (7 studies), spine (4 studies) and pelvic ring (4 studies). Most studies were retrospective while 5 being prospective.

## Main text

### Humeral fractures

In humeral fracture, is important to obtain a safe anchorage of the implants for early mobilization of the shoulder, avoiding protracted functional impairment and stiffness. Despite biomechanical innovations, the anchorage of the implants using screws is still the most frequent option and screws failure is the main responsible for global implant failure. Clinical trials report improvement in anchorage systems by using calcium phosphate cement to fill the gap in case of humeral head bone loss [23]. Cannulated screws in combination with angular stable plates enhance the fracture fixation and allow the insertion of PMMA cement [24]. Load cycle, varus bending, and torsional stress tests demonstrate that the use of PMMA in humeral fractures enhance the healing process. A biomechanical study showed that augmentation was most effective in low bone quality and not effective in good bone quality. In conclusion, augmentation had a good clinical impact if the starting bone quality is not good [25]. Bone quality is not uniformly distributed in the humeral head explaining how it is still unclear which type and how many screws are necessary to achieve a beneficial augmentation. A study has been conducted for evaluating local bone quality in the humeral head by measuring the breakaway torque at the screw tip: screws placed in the anteromedial and anteroinferior aspects of the head showed the lowest breakaway torques and were selected for augmentation with 0.5 ml of PMMA cement, achieving almost the same stability as augmentation of four most proximal screws [26].

### Hip fractures

Hip fractures are still associated with significant morbidity and mortality and a major health problem. The epidemiological data varies between countries, but it is globally estimated that hip fractures affect around 18% of women and 6% of men, up to an expected number of 6.3 million per year in 2050 [27, 28]. The direct costs associated with this condition are enormous since it requires a long period of hospitalization and subsequent rehabilitation. Given the importance of maintaining function and independence in the geriatric patient population, the use of PMMA for augmentation of fixation in hip fractures is of growing interest. The use of bone cement augmentation has been reportedly used for plate, screw, and nail osteosynthesis in elderly patients. In fact, a few studies demonstrated an increased bone-implant interface, improved implant anchorage, reduced screw cut-out, and improved early full-weight bearing when PMMA augmentation was adopted for hip fractures [29–31].

Femoral lateral fractures treated by nail and cephalic hydroxyapatite coated screws showed higher mechanical stability due to improved implant osteointegration demonstrated using dual X-ray absorptiometry (DeXa) exam [32]. The treatment of trochanteric fractures with a dynamic hip screw (DHS) augmented with PMMA or a resorbable bone cement based on calcium phosphate has shown greater biomechanical strength, faster pain reduction, and improved healing compared to a control group [33]. In a clinical prospective study for proximal femur fractures, treatment with a PMMA-augmented DHS showed good fracture consolidation without any adverse complications such as avascular necrosis of the femoral head [34]. Some authors treated patients with trochanteric fractures using antirotational proximal femur nail (PFNA) and subsequent injection of cement into the fracture line using a trauma needle kit into the spiral blade. According to this study, the injection allows earlier patient mobilization and the subsequent daily activities with a satisfaction almost comparable with the life of the patient prior to trauma [35]. A recent study comparing post-surgical weight bearing while walking highlighted early mobilization and higher weight bearing in augmented patients for femoral fracture [36]. A study by Sermon et al. compared augmented PFNA in cadaveric femoral head, mimicking an intertrochanteric fracture showing increased rates of resistance to compressive cyclic loading compared to non-augmented fractures [37]. Other studies confirm that using this special high viscosity bone cement applied via a PFNA blade, augmentation can be safely and effectively achieved using similar standard implantation techniques to the non-augmented device [19, 38]. Instead of the conventional spiral blade, a perforated spiral blade is used in cement augmented PFNA nailing to better achieve cement dissemination into the femoral head.

Beware of the possibility of intraarticular leakage into the hip joint while evaluating the fracture treatment. A study by Schuetze and colleagues on 152 patients showed a zero leakage rate while preventing mechanical screw cut-out. Furthermore, a very low complication rate was reported but for sudden blood pressure drop (a fact expected when doing augmentation) [19]. Approximately 3–5 ml of cement should be injected via the blade, as to not exceed a maximum volume of 6 ml; cement hardening takes about 10–15 min [39].

### Distal tibial fractures

Tibial fractures count for about 3% of all fractures. In the elderly population alone, this percentage reaches 10%. Typically, distal tibial fractures occur as a direct axial compressive force is applied in conjunction with a valgus force over the lower leg. The surgical treatment must reestablish joint congruency, restoring range of motion, alignment, and stability. To reduce the risk of ankle and knee arthritis, the surgeon must correct any depression of the joint surfaces. The classic method to avoid articular fragment depression is filling the subchondral space with autologous or allogenic bone transplant; both solutions offer weak results in the immediate post-surgery, demanding weight-bearing restriction during the healing process, to avoid secondary fractures and/or pseudoarthrosis [40–43]. To substitute bone transplants, various biomaterials have been introduced for filling the subchondral spaces in tibial plateau fractures. These materials are frequently available as preformed blocks that can be used to fill bone defects intraoperatively because they are injectable and self-hardening. The compressive strength and the material hardness are the mechanical properties most often used to characterize the mechanical behavior of a bone graft substitute. If a material is too hard, the mechanical environment for the overlying cartilage might be negatively affected. It is important to consider that a fracture site is also subject to shear and bending forces. When using materials with low bending and shear resistance, it is necessary to use screws or hardware that can neutralize these forces to provide a mechanical construct that can withstand not only compression forces, but also shear and bending forces. Injectable ceramic biphasic bone substitute was used to fill residual void in tibial plateau fractures treated by percutaneous or open reduction and interna fixation (ORIF) technique with a good clinical and radiological outcome in terms of articular joint alignment and knee function score [44]. Bioresorbable calcium phosphate cement placed in the defect cavity for subarticular support represent a good choice in terms of prevention of subsidence [45] even if some authors reported higher fatigue strength and ultimate load that autogenous bone graft repairs [46].

### Spine fractures

Cement leakage is the most frequent complication after vertebroplasty and kyphoplasty procedures. Different studies showed that increased amounts of PMMA injected during procedures such as vertebroplasty and kyphoplasty are associated with higher stiffness, higher risk of cement leakage and potential exothermal damage while not improving clinical outcome [47]. Some studies focus on the optimal quantity of cement injected that should be the least amount needed for clinical efficacy, approximately corresponding to 15% of the vertebral volume to be treated [48]. Several factors are associated with a lower risk of cement leakage: balloons inflation prior to cement injection, the employ of large-diameter needles to keep injection pressure low, the use of high viscosity cement, and to visualize the injected area with high-quality imaging techniques. Particularly, balloon catheter is used to prepare the fractured zone for PMMA injection, reducing cement leakage. A development of this technique is the radiofrequency-targeted vertebral augmentation which leads to comparable result for augmentation and pain relief [49]. Augmentation procedures for vertebral pedicles include a cement mantle between pedicle screw and cancellous bone, allowing for undisturbed polymerization of the cement mantle until plastic cement deformation is no longer present. In larger spinal defects (e.g., after gross resections or when filling metallic cages), the benefits of using biocompatible/degradable cements may be limited, considering the large distances and volumes involved with respect to potential vascular ingrowth necessary for bone remodeling and creeping substitution. In preventing spinous process fractures after interspinous spacer implants in patients with risk factors for fragility fractures, posterior vertebral arch augmentation (spinoplasty) seems effective [50].

### Pelvic ring fractures

Current literature shows how the absolute number of pelvic fractures is continuously rising due to the increasing aging of the population. In aging and osteoporosis, augmentation of the implant with bone cement during osteosynthesis seems to be an option to avoid secondary displacement [51]. Cement augmentation is known to significantly increases the fixation strength in iliosacral screw osteosynthesis [51, 52] with higher stiffness and pullout force and reduced screw loosening [53]. Recently, the cement-augmented transiliacal internal fixator (caTIFI) has been adopted in fragility fractures of the pelvis. In this technique, the Schanz screws applied to the ilium were placed in an oblique dorsoventral direction into the supraacetabular bone canal, while checking the correct position of the screws before implantation [54].

## Conclusion

There is a relative lack of knowledge about the role of augmentation in surgical treatment of fragility fractures.

The limit of our study lies in the fact that we analyzed different augmentation techniques dividing them based on the anatomical site of the fracture. A subdivision by single fracture type would have been more accurate but the lack of sufficient data would have reduced the scientific validity of further subdivision into subgroups.

However, to answer our opening question, our literature review finally shows that different treatment options are currently available for augmentation in fragility fracture osteosynthesis. Various materials can be used for reconstruction of bone defects in fragility fractures in different anatomic locations. There is no review comparing which type of augmentation technique could be superior in a specific anatomic location. Strengthening implant fixation using materials such as PMMA have shown promising mechanical and clinical results with good biocompatibility. PMMA is the most investigated and used augmentation technique and it is the only one to show significative clinical results.

In elderly patients, the need for early weightbearing and mobilization to avoid medical complication is of great importance. Therefore, new biomaterials that improve fixation in osteoporotic bone should be investigated. According to our findings, nowadays there are no recommendations and no consensus about the use of augmentation techniques in osteoporotic fractures. Additional studies are necessary to evaluate the mechanical, clinical, and biomedical aspects of augmentation and to provide guidelines. Also, lowering in-hospital stay and healthcare cost contribute as additional benefits. In this complex framework, we strongly encourage orthopedic surgeons to promote research around this relevant and current topic because it would be a great success to reach a global consensus to considerate augmentation technique in planning for elderly people with fragility fractures especially in comminuted and unstable patterns.

## Data Availability

The datasets used and/or analyzed during the current study available from the corresponding author on reasonable request.

## Abbreviations

BMD: bone mineral density
WHO: World health organization
PMMA: Polymethylmethacrylate
PRISMA: Preferred reporting items for systematic reviews and meta-analyses
DeXa: Dual X-ray absorptiometry
DHS: Dynamic hip screw
PFNA: Proximal femur nail antirotation
ORIF: Open reduction and interna fixation
caTIFI: Cement-augmented transiliacal internal fixation.
